# Bullous dermatologic reactions in immune checkpoint inhibitor therapy: A systematic review

**DOI:** 10.1016/j.jdin.2025.08.015

**Published:** 2025-10-03

**Authors:** Chinathip Theerawattanawit, Patcharapong Rujirawan, Pawinee Rerknimitr

**Affiliations:** Division of Dermatology, Department of Medicine, Faculty of Medicine, Center of Excellence for Skin and Allergy Research, Chulalongkorn University, Bangkok, Thailand

**Keywords:** bullous reaction, cancer, checkpoint inhibitor therapy, immune checkpoint inhibitor

*To the Editor:* Immune checkpoint inhibitors (ICIs) have revolutionized cancer treatment by enhancing antitumor immunity but may cause immune-related adverse events, commonly affecting the skin. Immune-related cutaneous adverse events range from pruritus to bullous dermatologic reactions (BDRs), including bullous pemphigoid (BP) and severe conditions such as Stevens–Johnson syndrome (SJS) and toxic epidermal necrolysis (TEN). Although BP and SJS/TEN have been well reviewed, data on other ICI-induced BDRs (ICI-BDRs) remain limited.^1^^,^^2^ We conducted a systematic review to characterize the clinical features, diagnosis, management, and outcomes of ICI-BDRs.

Following Preferred Reporting Items for Systematic Reviews and Meta-Analyses guidelines, we systematically searched PubMed and Scopus from inception to September 9, 2024 (Fig 1). ICI-BDRs include BP, SJS/TEN, erythema multiforme (EM), pemphigus vulgaris, lichen planus pemphigoides, bullous lichen planus, mucous membrane pemphigoid, linear IgA bullous dermatosis, and dermatitis herpetiformis. We identified 2102 patients (pooled median age: 68 years) from 346 studies, with a male predominance (55.99%). The most common underlying malignancy was melanoma (*n =* 596, 28.35%), and anti-programmed cell death protein 1 therapy was most frequently used (*n =* 1507, 71.69%). Hepatitis was the most co-occurring immune-related adverse event (*n =* 46, 2.19%). Full baseline characteristics are shown in Supplementary Table I (available via Mendeley at https://data.mendeley.com/datasets/kzkp5v3rvx/1).

**Fig 1:**
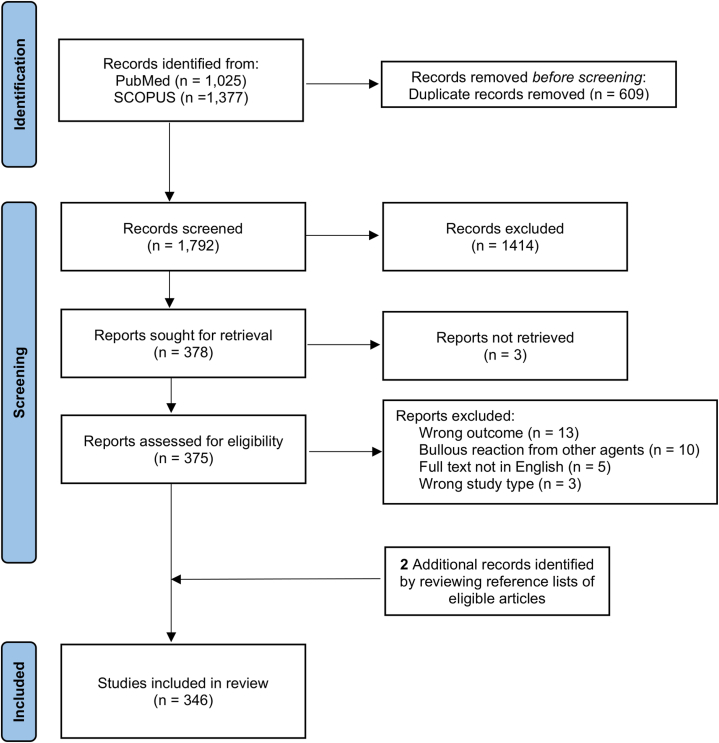
A flow diagram of the literature screened using the Preferred Reporting Items for Systematic Reviews and Meta-Analyses guidelines.

Case summaries are presented in Table I. For BP (*n =* 1142), the pooled median latency was 210 days. Prodromal symptoms, such as urticaria, occurred in 16%, and mucosal involvement was reported in 8.49%. Among 590 patients with SJS/TEN, the pooled median latency was 24 days, which appeared to be higher than that reported in the literature.^3^ The median (IQR) Severity-of-Illness Score for Toxic Epidermal Necrolysis (SCORTEN) score was 3(1), with the pooled median affected body surface area of 16.5%. Mucosal involvement occurred in 20.17%, most commonly affecting the oral mucosa, followed by the ocular and genitalia. For EM (*n =* 270), the pooled median latency was 30 days, with oral mucosa being the most frequently involved.

**Table I:** Clinical and serologic characteristics, immune checkpoint inhibitor adjustment, disease outcomes, and treatment of each ICI-induced bullous dermatologic reaction

Characteristics	BP (*n =* 1142)	SJS/TEN (*n =* 590)	EM (*n =* 270)	PV (*n =* 43)	LPP (*n =* 26)
**Clinical characteristics**					
Age in years∗	70 (70-74)	64 (34-64)	66 (10)	62 (23)	64.5 (18)
Sex					
⋅ Male, *n* (%)	722 (63.22%)	307 (52.03%)	133 (49.26%)	6 (13.95%)	9 (33.33%)
⋅ Female, *n* (%)	222 (19.44%)	190 (32.2%)	85 (31.48%)	2 (4.65%)	18 (66.67%)
⋅ Not reported, *n* (%)	198 (17.34%)	93 (15.76%)	52 (19.26%)	35 (81.4%)	0 (0%)
Latency period (d)†	210 (204-287.7)	24 (21-63)	30 (30-66.5)	126 (171)	120 (112.5)
Prodromal rash					
⋅ Present, *n* (%)	188 (16.46%)	N/A	N/A	N/A	N/A
Urticaria, *n* (%)	115 (10.07%)	N/A	N/A	N/A	N/A
Eczema, *n* (%)	24 (2.1%)	N/A	N/A	N/A	N/A
⋅ Absent, *n* (%)	73 (6.39%)	N/A	N/A	N/A	N/A
⋅ Not reported, *n* (%)	881 (77.15%)	N/A	N/A	N/A	N/A
Mucosal involvement					
⋅ Present, *n* (%)	97 (8.49%)	119 (20.17%)	9 (3.33%)	4 (9.3%)	10 (38.46%)
Eye involvement, *n* (%)	N/A	39 (6.61%)	1 (0.37%)	0 (0%)	0 (0%)
Oral involvement, *n* (%)	N/A	76 (12.88%)	8 (2.96%)	4 (9.3%)	10 (38.46%)
Genital involvement, *n* (%)	N/A	25 (4.24%)	2 (0.74%)	0 (0%)	1 (3.85%)
Laryngeal involvement, *n* (%)	N/A	N/A	N/A	N/A	N/A
Nasal involvement, *n* (%)	N/A	N/A	N/A	N/A	N/A
⋅ Absent, *n* (%)	289 (25.31%)	12 (2.03%)	9 (3.33%)	3 (6.98%)	15 (55.56%)
⋅ Not reported, *n* (%)	756 (66.2%)	459 (77.8%)	252 (93.33%)	36 (83.72%)	1 (3.7%)
SCORTEN, median (IQR)	N/A	3 (1)	N/A	N/A	N/A
BSA (%), pooled median (95% CI)	N/A	16.5 (16.5-35)	N/A	N/A	N/A
Palm involvement, *n* (%)	N/A	N/A	5 (1.85%)	N/A	N/A
Plantar involvement, *n* (%)	N/A	N/A	3 (1.11%)	N/A	N/A
Nail involvement, *n* (%)	N/A	N/A	N/A	N/A	N/A
GI symptoms, *n* (%)	N/A	N/A	N/A	N/A	N/A
**Serologic characteristics**					
Positive for anti-BPAG1, n (%)	30 (2.63%)	N/A	N/A	N/A	0 (0%)
Positive for anti-BPAG2, n (%)	134 (11.12%)	N/A	N/A	N/A	12 (46.15%)
Positive for Anti-Dsg 1, *n* (%)	N/A	N/A	N/A	4 (9.3%)	N/A
Positive for Anti-Dsg 3, *n* (%)	N/A	N/A	N/A	6 (13.95%)	N/A
**ICI adjustment**					
⋅ Discontinued,					
From irCAEs, *n* (%)	469 (41.07%)	323 (54.75%)	114 (42.22%)	3 (6.98%)	16 (61.54%)
From other causes, *n* (%)	54 (4.72%)	0 (0%)	2 (0.74%)	2 (4.65%)	6 (23.07%)
⋅ Continued, *n* (%)	170 (14.89%)	6 (1.02%)	35 (12.96%)	1 (2.33%)	2 (7.69%)
⋅ Temporary discontinued then continued after rash improvement, *n* (%)	53 (4.64%)	1 (0.17%)	1 (0.37%)	0 (0%)	1 (3.85%)
⋅ Not reported, *n* (%)	396 (34.68%)	260 (44.07%)	118 (43.70%)	37 (86.05%)	1 (3.85%)
**Rash outcome**					
⋅ Remission, *n* (%)	292 (25.57%)	111 (18.81%)	16 (5.93%)	7 (16.28%)	24 (92.31%)
⋅ Partial response, *n* (%)	91 (7.97%)	7 (1.19%)	1 (0.37%)	0 (0%)	1 (3.85%)
⋅ Refractory, *n* (%)	13 (1.14%)	0 (0%)	0 (0%)	0 (0%)	0 (0%)
⋅ Death, *n* (%)	0 (0%)	21 (3.56%)	0 (0%)	0 (0%)	0 (0%)
⋅ Not reported, *n* (%)	746 (65.32%)	451 (76.44%)	253 (93.70%)	36 (83.72%)	1 (3.85%)
**Cancer outcome**					
⋅ Complete remission, *n* (%)	89 (7.79%)	4 (0.68%)	0 (0%)	1 (2.33%)	4 (15.38%)
⋅ Partial response, *n* (%)	60 (5.26%)	6 (1.02%)	2 (0.74%)	1 (2.33%)	0 (0%)
⋅ Stable disease, *n* (%)	81 (7.09%)	6 (1.02%)	0 (0%)	0 (0%)	3 (11.54%)
⋅ Progression, *n* (%)	74 (6.48%)	13 (2.20%)	5 (1.85%)	4 (9.30%)	8 (30.77%)
⋅ Death, *n* (%)	5 (0.44%)	22 (3.73%)	0 (0%)	0 (0%)	0 (0%)
⋅ Not reported, *n* (%)	833 (72.94%)	539 (91.36%)	263 (97.41%)	37 (86.05%)	11 (42.31%)
**Treatment**					
⋅ Topical steroid, *n* (%)	106 (9.28%)	2 (0.34%)	2 (0.74%)	2 (4.65%)	4 (15.38%)
⋅ Systemic steroid, *n* (%)	264 (23.12%)	137 (23.22%)	14 (5.19%)	4 (9.30%)	21 (80.77%)
⋅ Nonsteroid immunosuppressive agents, *n* (%)	97 (8.49%)	13 (2.20%)	3 (1.11%)	1 (2.33%)	5 (19.23%)
⋅ Cyclosporine, *n* (%)	1 (0.09%)	13 (2.20%)	0 (0%)	0 (0%)	0 (0%)
⋅ Azathioprine, *n* (%)	3 (0.26%)	0 (0%)	1 (0.37%)	0 (0%)	0 (0%)
⋅ Mycophenolate mofetil, *n* (%)	5 (0.44%)	1 (0.17%)	0 (0%)	0 (0%)	0 (0%)
⋅ Methotrexate, *n* (%)	11 (0.96%)	0 (0%)	0 (0%)	1 (2.33%)	1 (3.85%)
⋅ Sulfasalazine, *n* (%)	1 (0.09%)	0 (0%)	0 (0%)	0 (0%)	0 (0%)
⋅ Tetracycline antibiotics, *n* (%)	95 (8.32%)	0 (0%)	2 (0.74%)	0 (0%)	2 (7.69%)
⋅ Dapsone, *n* (%)	17 (1.49%)	0 (0%)	0 (0%)	0 (0%)	2 (7.69%)
⋅ Hydroxychloroquine, *n* (%)	0 (0%)	0 (0%)	0 (0%)	0 (0%)	0 (0%)
⋅ Retinoid, *n* (%)	0 (0%)	0 (0%)	0 (0%)	0 (0%)	0 (0%)
⋅ Niacinamide, *n* (%)	40 (3.5%)	0 (0%)	0 (0%)	0 (0%)	1 (3.85%)
⋅ Biologics, *n* (%)	41 (3.59%)	26 (4.41%)	0 (0%)	1 (2.33%)	0 (0%)
⋅ Rituximab, *n* (%)	20 (1.75%)	1 (0.17%)	0 (0%)	1 (2.33%)	0 (0%)
⋅ Dupilumab, *n* (%)	12 (1.05%)	0 (0%)	0 (0%)	0 (0%)	0 (0%)
⋅ Omalizumab, *n* (%)	10 (0.88%)	0 (0%)	0 (0%)	0 (0%)	0 (0%)
⋅ Adalimumab, *n* (%)	0 (0%)	1 (0.17%)	0 (0%)	0 (0%)	0 (0%)
⋅ Etanercept, *n* (%)	0 (0%)	12 (2.03%)	0 (0%)	0 (0%)	0 (0%)
⋅ Infliximab, *n* (%)	0 (0%)	11 (1.86%)	0 (0%)	0 (0%)	0 (0%)
⋅ Other biologics, *n* (%)	0 (0%)	1 (0.17%)	0 (0%)	0 (0%)	0 (0%)
⋅ IVIg, *n* (%)	14 (1.23%)	62 (10.51%)	0 (0%)	0 (0%)	0 (0%)
⋅ Plasma exchange, *n* (%)	2 (0.18%)	4 (0.68%)	0 (0%)	1 (2.33%)	0 (0%)
⋅ Other, *n* (%)	5 (0.44%)	5 (0.85%)	1 (0.37%)	0 (0%)	0 (0%)

Characteristics	Bullous LP (*n =* 13)	MMP (*n =* 11)	LABD (*n =* 5)	DH (*n =* 2)
**Clinical characteristics**				
Age in years∗	74.5 (7.5)	71 (20)	56 (4)	49.5 (45)
Sex				
⋅ Male, *n* (%)	2 (15.38%)	5 (45.45%)	2 (40%)	1 (50%)
⋅ Female, *n* (%)	5 (38.46%)	5 (45.45%)	2 (40%)	1 (50%)
⋅ Not reported, *n* (%)	6 (46.15%)	1 (9.09%)	1 (20%)	0 (0%)
Latency period (d)†	84 (96)	174 (305)	97 (166)	420 (780)
Prodromal rash				
⋅ Present, *n* (%)	N/A	N/A	N/A	N/A
Urticaria, *n* (%)	N/A	N/A	N/A	N/A
Eczema, *n* (%)	N/A	N/A	N/A	N/A
⋅ Absent, *n* (%)	N/A	N/A	N/A	N/A
⋅ Not reported, *n* (%)	N/A	N/A	N/A	N/A
Mucosal involvement				
⋅ Present, *n* (%)	3 (23.08%)	11 (100%)	1 (20%)	0 (0%)
Eye involvement, *n* (%)	N/A	2 (18.18%)	1 (20%)	N/A
Oral involvement, *n* (%)	3 (23.08%)	10 (90.91%)	0 (0%)	N/A
Genital involvement, *n* (%)	N/A	1 (3.7%)	0 (0%)	N/A
Laryngeal involvement, *n* (%)	N/A	2 (18.18%)	N/A	N/A
Nasal involvement, *n* (%)	N/A	2 (18.18%)	N/A	N/A
⋅ Absent, *n* (%)	0 (0%)	0 (0%)	4 (80%)	1 (50%)
⋅ Not reported, *n* (%)	10 (76.92%)	0 (0%)	0 (0%)	1 (50%)
SCORTEN, median (IQR)	N/A	N/A	N/A	N/A
BSA (%), pooled median (95%CI)	N/A	N/A	N/A	N/A
Palm involvement, *n* (%)	N/A	N/A	N/A	N/A
Plantar involvement, *n* (%)	N/A	N/A	N/A	N/A
Nail involvement, *n* (%)	0 (0%)	N/A	N/A	N/A
GI symptoms, *n* (%)	N/A	N/A	N/A	N/A
**Serologic characteristics**				
Positive for anti-BPAG1, *n* (%)	N/A	N/A	N/A	N/A
Positive for anti-BPAG2, *n* (%)	N/A	N/A	N/A	N/A
Positive for Anti-Dsg 1, *n* (%)	N/A	N/A	N/A	N/A
Positive for Anti-Dsg 3, *n* (%)	N/A	N/A	N/A	N/A
**ICI adjustment**				
⋅ Discontinued,				
From irCAEs, *n* (%)	6 (46.15%)	6 (54.55%)	1 (20%)	0 (0%)
From other causes, *n* (%)	0 (0%)	0 (0%)	0 (0%)	0 (0%)
⋅ Continued, *n* (%)	1 (7.69%)	3 (27.27%)	2 (40%)	2 (100%)
⋅ Temporary discontinued then continued after rash improvement, *n* (%)	1 (7.69%)	0 (0%)	0 (0%)	0 (0%)
⋅ Not reported, *n* (%)	5 (38.46%)	2 (18.18%)	2 (40%)	0 (0%)
**Rash outcome**				
⋅ Remission, *n* (%)	7 (53.85%)	8 (72.73%)	3 (60%)	1 (50%)
⋅ Partial response, *n* (%)	1 (7.69%)	3 (27.27%)	0 (0%)	0 (0%)
⋅ Refractory, *n* (%)	0 (0%)	0 (0%)	0 (0%)	0 (0%)
⋅ Death, *n* (%)	0 (0%)	0 (0%)	0 (0%)	0 (0%)
⋅ Not reported, *n* (%)	5 (38.46%)	0 (0%)	2 (40%)	1 (50%)
**Cancer outcome**				
Complete remission, *n* (%)	0 (0%)	2 (18.18%)	0 (0%)	2 (100%)
Partial response, *n* (%)	1 (7.69%)	1 (9.09%)	0 (0%)	0 (0%)
Stable disease, *n* (%)	2 (15.38%)	1 (9.09%)	0 (0%)	0 (0%)
Progression, *n* (%)	3 (23.08%)	1 (9.09%)	2 (40%)	0 (0%)
Death, *n* (%)	0 (0%)	0 (0%)	0 (0%)	0 (0%)
Not reported, *n* (%)	7 (53.85%)	6 (54.55%)	3 (60%)	0 (0%)
**Treatment**				
⋅ Topical steroid, *n* (%)	2 (15.38%)	2 (18.18%)	0 (0%)	1 (50%)
⋅ Systemic steroid, *n* (%)	5 (38.46%)	4 (36.36%)	2 (40%)	0 (0%)
⋅ Nonsteroid immunosuppressive agents, *n* (%)	1 (7.69%)	6 (54.55%)	2 (40%)	0 (0%)
⋅ Cyclosporine, *n* (%)	0 (0%)	0 (0%)	0 (0%)	0 (0%)
⋅ Azathioprine, *n* (%)	0 (0%)	0 (0%)	0 (0%)	0 (0%)
⋅ Mycophenolate mofetil, *n* (%)	0 (0%)	0 (0%)	0 (0%)	0 (0%)
⋅ Methotrexate, *n* (%)	0 (0%)	1 (9.09%)	0 (0%)	0 (0%)
⋅ Sulfasalazine, *n* (%)	0 (0%)	0 (0%)	0 (0%)	0 (0%)
⋅ Tetracycline antibiotics, *n* (%)	0 (0%)	4 (36.36%)	1 (20%)	0 (0%)
⋅ Dapsone, *n* (%)	0 (0%)	1 (9.09%)	2 (40%)	0 (0%)
⋅ Hydroxychloroquine, *n* (%)	1 (7.69%)	0 (0%)	0 (0%)	0 (0%)
⋅ Retinoid, *n* (%)	1 (7.69%)	0 (0%)	0 (0%)	0 (0%)
⋅ Niacinamide, *n* (%)	0 (0%)	1 (9.09%)	0 (0%)	0 (0%)
⋅ Biologics, *n* (%)	0 (0%)	2 (18.18%)	0 (0%)	0 (0%)
⋅ Rituximab, *n* (%)	0 (0%)	2 (18.18%)	0 (0%)	0 (0%)
⋅ Dupilumab, *n* (%)	0 (0%)	0 (0%)	0 (0%)	0 (0%)
⋅ Omalizumab, *n* (%)	0 (0%)	0 (0%)	0 (0%)	0 (0%)
⋅ Adalimumab, *n* (%)	0 (0%)	0 (0%)	0 (0%)	0 (0%)
⋅ Etanercept, *n* (%)	0 (0%)	0 (0%)	0 (0%)	0 (0%)
⋅ Infliximab, *n* (%)	0 (0%)	0 (0%)	0 (0%)	0 (0%)
⋅ Other biologics, *n* (%)	0 (0%)	0 (0%)	0 (0%)	0 (0%)
⋅ IVIg, *n* (%)	0 (0%)	1 (9.09%)	0 (0%)	0 (0%)
⋅ Plasma exchange, *n* (%)	0 (0%)	0 (0%)	0 (0%)	0 (0%)
⋅ Other, *n* (%)	0 (0%)	0 (0%)	0 (0%)	1 (50%)

*BP*, Bullous pemphigoid; *BSA*, body surface area; *DH*, dermatitis herpetiformis; *EM*, erythema multiforme; *GI*, gastrointestinal; *ICI*, immune checkpoint inhibitor; *irCAE*, immune-related cutaneous adverse event; *IVIg*, intravenous immunoglobulin; *LP*, lichen planus; *LPP*, lichen planus pemphigoides; *LABD*, linear IgA bullous dermatosis; *MMP*, mucous membrane pemphigoid; *N/A*, not applicable; *PV*, pemphigus vulgaris; SCORTEN, Severity-of-Illness Score for Toxic Epidermal Necrolysis; *SJS*, Stevens–Johnson syndrome; *TEN*, toxic epidermal necrolysis.

∗ The age of patients with BP and SJS/TEN is presented as a pooled median (95% CI), while that of other groups is shown as median (IQR).

† The latency period for patients with BP, SJS/TEN, and EM is presented as pooled median (95% CI), while that of other groups is shown as a median (IQR).

Among reported cases, ICIs were commonly discontinued following the onset of ICI-BDRs, with SJS/TEN showing the highest discontinuation rate (97.88%), in line with the National Comprehensive Cancer Network guidelines classifying SJS/TEN as grade ≥3 immune-related adverse events requiring permanent discontinuation.^4^ Rechallenge with ICIs was infrequently reported in BP, SJS, EM, lichen planus pemphigoides, and bullous lichen planus cases; notably, 1 patient with SJS/TEN successfully underwent ICI rechallenge.^5^ Immunosuppressive agents were the primary treatment, with systemic corticosteroids as the mainstay, except in dermatitis herpetiformis. Most BDRs (excluding BP and SJS/TEN) showed partial response or remission. Among SJS/TEN cases, 22 patients (3.73%) died, with 21 deaths attributed to the cutaneous eruption rather than the underlying malignancy. Details on histopathology are shown in Supplementary Table II (available via Mendeley at xxx).

Our findings underscore the diverse clinical spectrum and serious implications of ICI-BDRs, warranting heightened vigilance, early recognition, and multidisciplinary management to optimize both dermatologic and oncologic outcomes.

## Conflicts of interest

None disclosed.
